# Associations Between Stress, Health Behaviors, and Quality of Life in Young Couples During the Transition to Survivorship: Protocol for a Measurement Burst Study

**DOI:** 10.2196/53307

**Published:** 2024-04-23

**Authors:** Dalnim Cho, Michael Roth, Susan K Peterson, Kristofer Jennings, Seokhun Kim, Shiao-Pei Weathers, Sairah Ahmed, J Andrew Livingston, Carlos Barcenas, Y Nancy You, Kathrin Milbury

**Affiliations:** 1 Department of Health Disparities Research The University of Texas MD Anderson Cancer Center Houston, TX United States; 2 Department of Pediatrics The University of Texas MD Anderson Cancer Center Houston, TX United States; 3 Department of Behavioral Science The University of Texas MD Anderson Cancer Center Houston, TX United States; 4 Department of Biostatistics The University of Texas MD Anderson Cancer Center Houston, TX United States; 5 The Center for Clinical Research and Evidence-Based Medicine The University of Texas McGovern Medical School Houston, TX United States; 6 Department of Neuro-Oncology The University of Texas MD Anderson Cancer Center Houston, TX United States; 7 Department of Lymphoma–Myeloma The University of Texas MD Anderson Cancer Center Houston, TX United States; 8 Department of Sarcoma Medical Oncology The University of Texas MD Anderson Cancer Center Houston, TX United States; 9 Department of Breast Medical Oncology The University of Texas MD Anderson Cancer Center Houston, TX United States; 10 Department of Colon & Rectal Surgery The University of Texas MD Anderson Cancer Center Houston, TX United States

**Keywords:** young adult survivors, caregivers, dyadic, couple-based, stress, health behaviors, quality of life, transition to survivorship, measurement-burst

## Abstract

**Background:**

Cancer is a life-threatening, stressful event, particularly for young adults due to delays and disruptions in their developmental transitions. Cancer treatment can also cause adverse long-term effects, chronic conditions, psychological issues, and decreased quality of life (QoL) among young adults. Despite numerous health benefits of health behaviors (eg, physical activity, healthy eating, no smoking, no alcohol use, and quality sleep), young adult cancer survivors report poor health behavior profiles. Determining the associations of stress (either cancer-specific or day-to-day stress), health behaviors, and QoL as young adult survivors transition to survivorship is key to understanding and enhancing these survivors’ health. It is also crucial to note that the effects of stress on health behaviors and QoL may manifest on a shorter time scale (eg, daily within-person level). Moreover, given that stress spills over into romantic relationships, it is important to identify the role of spouses or partners (hereafter partners) in these survivors’ health behaviors and QoL.

**Objective:**

This study aims to investigate associations between stress, health behaviors, and QoL at both within- and between-person levels during the transition to survivorship in young adult cancer survivors and their partners, to identify the extent to which young adult survivors’ and their partners’ stress facilitates or hinders their own and each other’s health behaviors and QoL.

**Methods:**

We aim to enroll 150 young adults (aged 25-39 years at the time of cancer diagnosis) who have recently completed cancer treatment, along with their partners. We will conduct a prospective longitudinal study using a measurement burst design. Participants (ie, survivors and their partners) will complete a daily web-based survey for 7 consecutive days (a “burst”) 9 times over 2 years, with the bursts spaced 3 months apart. Participants will self-report their stress, health behaviors, and QoL. Additionally, participants will be asked to wear an accelerometer to assess their physical activity and sleep during the burst period. Finally, dietary intake (24-hour diet recalls) will be assessed during each burst via telephone by research staff.

**Results:**

Participant enrollment began in January 2022. Recruitment and data collection are expected to conclude by December 2024 and December 2026, respectively.

**Conclusions:**

To the best of our knowledge, this will be the first study that determines the interdependence of health behaviors and QoL of young adult cancer survivors and their partners at both within- and between-person levels. This study is unique in its focus on the transition to cancer survivorship and its use of a measurement burst design. Results will guide the creation of a developmentally appropriate dyadic psychosocial or behavioral intervention that improves both young adult survivors’ and their partners’ health behaviors and QoL and potentially their physical health.

**International Registered Report Identifier (IRRID):**

DERR1-10.2196/53307

## Introduction

### Overview

Each year, more than 80,000 adolescents and young adults (aged 15-39 years) are newly diagnosed with cancer in the United States [1], and cancer is the leading cause of disease-related death among adolescents and young adults [2]. Mortality rates in adolescent and young adult patients with cancer have decreased over the past decade, and the 5-year relative survival rate for adolescent and young adult patients with cancer is 85.5% [3]. However, this high relative survival rate may mask the true burden of cancer among adolescent and young adult survivors. Although cancer and its treatment can be a traumatic event for any individual, it may be particularly so for adolescents and young adults because cancer can cause delays and disruptions in their successful developmental transitions across various domains of life: biological or physical (eg, cancer can cause hair loss, infertility, and weight change), psychological (eg, lack of control over life and disruption in progress toward life goals), educational or occupational (eg, disruption in education and unemployment), social or relationship (eg, problems in relationship with significant other and interference with plans for having children), and financial (eg, large debt and bankruptcy) [4-13]. Cancer and its treatment can also cause adverse long-term effects among adolescents and young adults (eg, premature or accelerated aging, cardiotoxicity, and second malignant neoplasms) [14,15] and heightened risk of chronic conditions (eg, cardiovascular disease, hypertension, diabetes, and asthma) [14,16,17], psychological issues (eg, depressive symptoms and fear of cancer recurrence), and lower quality of life (QoL) among adolescents and young adults compared with their peers without cancer [14,18].

Interest in and calls for research on adolescent and young adult cancer survivors have dramatically increased since 2006 when the Adolescent and Young Adult Oncology Progress Review Group published a report about the state of the science regarding cancer among adolescents and young adults [19]. However, adolescents and young adults affected by cancer still have vast unmet health care needs, including psychosocial care needs [7,20-23], and remain an understudied subgroup of patients with cancer. In addition, adolescent and young adult cancer survivors are often considered a single entity, and much of the existing data comes from studies that grouped adolescents and young adults together, although individuals in this age range may face different developmental tasks. Thus, the proposed study focuses on young adult survivors who were diagnosed with cancer at 25 to 39 years old [24], whose primary source of support is more likely to be romantic partners, whereas pediatric and adolescent survivors are more likely to depend on their parents. Due to a lack of existing studies specific to young adult survivors, the literature review below is based on studies among adolescent and young adult survivors.

### Stress and Health Behaviors Among Young Adult Survivors

#### Overview

Strong evidence exists that engaging in healthy behaviors (eg, physical activity, low sedentary behavior, healthy eating, no smoking, no alcohol use, and quality sleep) is critical for cancer survivors’ health, as it reduces the likelihood of cardiotoxicity, development of secondary cancers, cancer recurrence, and cancer-specific and all-cause mortality [25-28] and improves QoL [26,29]. However, young adult survivors report poor health behavior profiles. Specifically, young adult survivors report higher rates of unhealthy eating than their peers without cancer [14,16,30], and 26% of young adult survivors versus 18% of their peers without cancer report current smoking [14,16,30]. In addition, 50% to 90% of young adult survivors report at least some alcohol use [30], and 31% of young adult survivors report no leisure-time physical activity [14]. Further, approximately 40% of young adult survivors report experiencing sleep issues (eg, trouble falling or staying asleep in the previous week), and those who report difficulties with sleep also indicate higher levels of psychological distress [31].

While stress can substantially influence negative health outcomes [32] and poor health behaviors [33-35], it remains an understudied factor for young adult survivors’ health behaviors. At present, almost nothing is known regarding the association between stress and health behaviors among young adult survivors. To better understand the association between stress and health behaviors in this survivor population, there are three crucial factors to be considered: (1) the transition from active treatment to survivorship; (2) interdependence between survivors and family caregivers, especially partners; and (3) within-person variabilities in stress and health behaviors.

#### Transition From Active Treatment to Survivorship

Transitioning from active treatment to posttreatment care is critical to cancer survivors’ long-term health. However, the transition is challenging because survivors are often lost to systematic follow-up, and opportunities to effectively intervene are missed [36]. Many people finish their primary cancer treatment being unaware of their heightened health risks and are ill-prepared to manage their future health care needs [36]. In particular, young adult survivors struggle with the transition; with the abrupt cessation of intensive support (feelings of abandonment); and with challenges regarding returning to work, study, and social or recreational activities, which is complicated by ongoing disease or treatment impacts and a collective dearth of knowledge and resources on how to cope with these challenges during this time [37]. Thus, beyond cancer or treatment-specific stress (eg, symptom burden and concerns about cancer recurrence), stress regarding postcancer life adjustment needs to be addressed to better understand young adults’ survivorship outcomes.

#### Interdependence Between Survivors and Their Partners

Cancer is often considered a *“*we-disease*”* [38]; survivors and their families tend to react to cancer as an interdependent system in which they influence each other’s health. As an example of this interdependence, stress experienced by a cancer survivor or by their spouse or partner (hereafter partner) can affect the QoL of the other person in the relationship [38,39]. Beyond cancer care demands and stressors shared with the survivor (eg, concerns about cancer recurrence or progression), partners of survivors may encounter various stressors in their everyday lives (eg, work deadlines and social obligations) that can spill over into the relationship. Thus, neglect of partners’ stress and coping and failure to include partners in young adult survivorship interventions may provide only a partial understanding of QoL and the challenges of the transition to survivorship among young adult survivors who have partners. However, to date, to the best of our knowledge, none of the existing interventions developed for and tested among young adult survivors to enhance their psychosocial outcomes (eg, QoL and psychological symptoms) have targeted survivor-partner dyads [40].

#### Within-Person Variabilities in Stress and Health Behaviors

Although previous studies have significantly advanced our understanding of cancer survivorship issues among young adults, most of these studies were cross-sectional and therefore could not capture potential dynamic changes in stress, health behaviors, and QoL during the transition to survivorship. While a few prospective longitudinal studies in young adult survivors exist [41-44], assessment intervals tend to range between 6 and 12 months and assume that stress, health behaviors, and QoL are fairly stable in the short term. However, evidence indicates that an individual’s stress and health behaviors may vary across days of a week [45-53], which may further explain reported short-term (eg, daily) within-person variations in QoL [54]. Moreover, within-person variations in stress, health behaviors, and QoL may exist in survivor-partner dyads [45,46]. Thus, global, aggregated measures of stress, health behaviors, and QoL at the between-person level or over longer time scales at the within-person level (eg, “How much were you stressed out in the *last week*?”; “How much did you drink alcohol in the *past month*?”; and “How was your overall QoL in the *past year*?”) may fail to reveal subtle and dynamic changes in stress, health behaviors, and QoL that can occur over shorter time scales (eg, daily intervals) [55].

A measurement burst design can assess both the between- and within-person processes in stress, health behaviors, and QoL. The design incorporates bursts of intensive repeated assessment within a relatively short period of time (eg, daily) that are repeated longitudinally, over more widely spaced intervals (eg, every 3 months), which will enable us to investigate the interaction of within-person processes that transpire over different time intervals [56,57]. However, to date, no published studies have investigated the extent to which young adult survivors and their partners facilitate or hinder each other’s short- and long-term adjustment during the transition to survivorship using the measurement burst design.

### This Study

#### Overview

The overarching purpose of this study is to investigate associations between stress, health behaviors, and QoL at both within-person and between-person levels over the transition to survivorship in young adult survivors and their partners to identify the extent to which young adult survivors’ and their partners’ stress facilitates or hinders their own and each other’s health behaviors and QoL. To examine both within-person and between-person changes, we will implement a measurement burst design [56,57], in which participants will be asked to complete a daily survey for 7 consecutive days (a “burst”) 9 times over 2 years, with the bursts spaced 3 months apart. Thus, there will be 3 levels: day, burst (3-month interval), and person. We have the following specific aims.

#### Aim 1

Aim 1 is to determine the within-person effects of stress on health behaviors at both day and burst levels. We hypothesize that on days or during bursts when stress is high, individuals are more likely to report physical inactivity, unhealthy eating, poor sleep quality, smoking (current smokers only), or alcohol use (current drinkers only). We also hypothesize that on days or during bursts when partners’ stress is high, survivors are more likely to report these unhealthy behaviors, and vice versa.

#### Aim 2

Aim 2 is to determine the within-person, day-level, and burst-level effects of stress on QoL and the effects of health behaviors on QoL. We hypothesize that individual’s QoL will be high on days or during bursts when their stress is low, when they perform physical activity, and when their sleep quality is good. We also hypothesize that on days or during bursts when partners’ stress is low, when partners perform physical activity, and when partners’ sleep quality is good, survivors are more likely to report high QoL, and vice versa.

#### Exploratory Aims

The two exploratory aims are as follows: (1) identifying 2-way cross-level interaction effects between day- or burst-level and person-level stress on health behaviors and QoL—in other words, we will examine whether the extent to which individuals’ day- or burst-level stress influences their health behaviors and whether QoL is stable or varies depending on their average person-level stress; and (2) identifying 2-way cross-level interaction effects between day- or burst-level stress and person-level stable factors (eg, sex, race, age at diagnosis, and type of cancer) on health behaviors and QoL—that is, we will examine whether the extent to which individuals’ day- or burst-level stress influences their health behaviors and whether QoL is stable or varies depending on their person-level factors.

## Methods

### Participants and Procedures

We aim to enroll 150 young adult survivors and their partners (ie, 300 individuals). Cancer survivors will be eligible if they (1) were diagnosed with cancer for the first time (ie, no previous history of cancer); (2) were a young adult (aged 25-39 years) at the time of diagnosis; (3) are within 3 months after the completion of treatment (eg, surgery, chemotherapy, and radiation therapy) with curative intent; (4) have no further planned cancer treatment (surgery, chemotherapy, radiation therapy, and immunotherapy) except for hormone therapy; and (5) have a valid phone number and email address. Survivors will be excluded if they (1) do not have significant others and (2) are unable to read, write, or speak English. Partners will be eligible if they (1) are aged ≥18 years; (2) are spouses or romantic partners (either married or not) cohabitating with the young adult survivor; (3) self-identify as the current primary caregivers of the young adult survivors; (4) are able to read, write, and speak English; and (5) have a valid phone number and email address.

During the study period, partners may change (because of separation, divorce, etc). If so, the current (ie, newly identified) partner (identified by the survivor, if applicable) will be contacted after permission is obtained from the survivor, and if interested and eligible, will be enrolled upon providing informed consent. We will record these changes in the dyads. If a member of a dyad drops out of the study, the other member will be asked to complete the study to investigate survivors’ or partners’ changes in stress, health behaviors, and QoL. If a survivor’s cancer recurs during the 2-year study period, the dyad (ie, both the survivor and their partner) will be withdrawn from the study.

Cancer survivors will be recruited at The University of Texas MD Anderson Cancer Center. Potentially eligible survivors will be initially identified by research staff via electronic health records and will be contacted via telephone. Only those who are interested in the study will be further screened. If the survivor is eligible and interested, we will obtain the contact information of their partner for the partner’s eligibility screening. If the partner is eligible and interested, the survivor-partner dyad will provide consent remotely via teleconferencing (eg, Zoom). We will also contact young adult cancer support groups and organizations through their websites and social media platforms to recruit non–MD Anderson cancer survivors. Interested individuals can submit a study interest form, and our research staff will contact them for phone screening.

All study procedures will be conducted remotely. After signed electronic consent forms are obtained, both survivors and partners will be asked to individually complete a baseline assessment in the form of a survey questionnaire via Research Electronic Data Capture (REDCap; Vanderbilt University), a secure, web-based app with controlled access designed to support data capture for research studies. This retrospective survey includes demographics, health status, stress, health behaviors, and QoL and will take approximately 1 hour to complete.

The daily survey will also be designed with REDCap, and the first daily survey period (ie, burst) will start within 10 days after completing the baseline survey. All participants will receive a survey link at 6 PM once per day for 7 consecutive days via their email; the brief 5-minute survey can be completed on the participant’s electronic device (computer and smartphone). Survivors and partners will receive separate links and be asked to complete their daily reports in the evenings (before going to sleep) regarding stress, health behaviors, and QoL. Survivors and partners will be asked to complete assessments separately. Each survey link will be valid for only 12 hours. Thus, participants will not be able to submit data for a given day early, but they will be able to provide it the next early morning (until 6 AM). Regardless of when data are provided (that evening or the next morning), the questions will be specific to the person’s experiences on the day the survey link is received.

During the burst, both survivors and partners will be asked to wear a blinded accelerometer (ActiGraph GT9X Link) on their nondominant wrist for 7 days and to engage in their daily routines as normal. The accelerometer with a prepaid envelope will be sent to the participants’ residential address. After wearing the accelerometer for 7 days, participants will return the device using the envelope. Moreover, participants will be asked to choose 2 days (a day to recall a weekday’s diet and another day to recall a weekend day’s diet) over the 7 days when they are available for a dietary assessment (the Automated Self-Administered 24-hour Dietary Assessment Tool [58]). Participants will be told that research staff will call them on the selected days or times for the assessment.

Before each 7-day daily assessment period, participants will be asked to complete a retrospective survey similar to the baseline survey. We will mail out the accelerometer (with the prepaid envelope) in advance so that participants can wear the accelerometer and begin the 7-day daily survey without delays once they complete the retrospective survey.

### Measures

#### Retrospective Survey Measures

##### Demographic and Medical Factors and Other Descriptive Factors and Covariates

Both survivors and partners will be asked about demographic (eg, sex, age, level of education and income, and marital or relationship status) and health and health care information (eg, existing comorbidities, health insurance, and height and weight to calculate BMI). Tumor characteristics will be obtained from self-report and medical records (for MD Anderson patients). Financial toxicity will be assessed with the Comprehensive Score for Financial Toxicity instrument [59], which consists of 12 items scored on a 5-point Likert scale and reveals high internal consistency and test-retest reliability (Cronbach α=.90) in patients with cancer. Financial toxicity will be assessed for both survivors and partners. For partners, the caregiving burden will be assessed with the short form of the Zarit Burden Inventory, a 12-item measure [60], which is validated among cancer caregivers [61]. Each question is scored on a 5-point Likert scale from 0 (never) to 4 (almost always). High scores represent a higher burden.

##### Stress

All participants will be asked to identify the most stressful event in the past 30 days (open-ended question). Then, the 4-item Perceived Stress Scale [62] will be administered; participants will rate each item from 0 (never) to 4 (very often).

##### Health Behaviors

For both survivors and partners, self-reported physical activity will be assessed with the Godin Leisure-Time Questionnaire [63], a brief measure assessing aerobic exercise, which is widely used in diverse populations including those with cancer [64]. In addition, we will assess strength exercise (eg, weight lifting and circuit training) with 1 item from the Health Information National Trends Survey [65]. Smoking will be assessed with items adapted from the Patient-Reported Outcomes Measurement Information System (PROMIS) Smoking Initiative [66] and the Health Information National Trends Survey [65], which include smoking behaviors (eg, “Have you smoked at least 100 cigarettes in your entire life?” [yes/no]; if yes, “How old were you when you first started smoking?”; and “Have you ever used an e-cigarette, even one or two times?” [yes/no]). Alcohol use will be assessed with 2 items from the Alcohol Use Disorders Identification Test–Consumption [67] asking about alcohol use frequency and quantity. Eating behaviors will be assessed with the validated, National Cancer Institute Diet History Questionnaire-III, which consists of 135 food and beverage line items and 26 dietary supplement questions [68]. Sleep will be assessed with the Pittsburgh Sleep Quality Index [69], an 18-item self-rated questionnaire that assesses the quality of sleep and sleep disturbances.

##### Relationship

Relationship will be assessed with 1 item measuring transformation of motivation [70] (indicating motivation for disease management is transformed from self-centered [eg, cancer is my/your problem] to relationship-centered [eg, cancer is our problem]) and with the 5-item Emotional Intimacy Scale, which showed good internal consistency (0.88) and test-retest reliability (0.85) [71].

##### QoL Measurement

For young adult survivors, we will use the 30-item satisfaction scale from the Late Adolescence and Young Adulthood Survivorship-Related Quality of Life (LAYA-SRQL) [72]. The LAYA-SRQL measure comprises 10 domains, including existential/spirituality, coping, relationship, dependence, vitality, health care, education/career, fertility, intimacy/sexuality, and cognition/memory. Participants can endorse “not applicable” if any item is not relevant to them. To reduce participants’ burden, we will use 11 items (ie, approximately 1 item per domain). In addition, both survivors’ and partners’ QoL will be assessed with the 10-item PROMIS–global health (measuring physical function, pain, fatigue, emotional distress, and social health) [73] so that we can compare the level of QoL between survivors and partners.

#### Daily Survey Measures

Both survivors and partners will report the following.

##### Stress

Stress will be assessed by adapting a measure of daily recording of coping with everyday stressful events [74]. Specifically, participants will be asked to report the most stressful event or issue of the day in their own words. Then, the level of distress will be assessed with 1 item asking, “On a scale from 1 to 100 (where 100 is the maximum distress that you could imagine and 1 is a minor annoyance), how stressful would you rate this problem or situation?”

##### Health Behaviors

The objective level of physical activity will be assessed with the accelerometer, which has strong reliability and validity [75]. Participants will wear a blinded accelerometer on their nondominant wrist for 7 days to assess typical physical activity. The Godin Leisure-Time Questionnaire [63] and the strength exercise item [65] will be asked to assess self-reported physical activity. Dietary intake will be assessed via telephone (from research staff) on 2 preselected days with the Automated Self-Administered 24-hour Dietary Assessment Tool [58], which produces a healthy eating index. The accelerometer will also objectively assess sleep (eg, total sleep time, sleep percentage, and wake after sleep onset). Thus, subjective sleep quality (over the last night) will be assessed with 1 item (“How would you rate your last night’s sleep quality overall?”) adapted from the Pittsburgh Sleep Quality Index [69]. Daily smoking (yes or no) will be assessed only among current smokers. Daily alcohol use (yes or no) and number of drinks will be assessed only among current drinkers. All of these self-reported measures will be assessed among both survivors and partners, adapting the aforementioned measures with the stem “TODAY, ...”

##### Relationship

The aforementioned 1 item measuring transformation of motivation [70] and 3 items from the Emotional Intimacy Scale [71] will be assessed with the reference “TODAY.”

##### QoL Measurement

We will assess survivors’ QoL with the same 11 items from the LAYA-SRQL. Survivors will be asked to answer each item with the stem “Please indicate the extent to which you are satisfied with this aspect of your life TODAY.” Participants can endorse “not applicable” if any item is not relevant to them. For both survivors and partners, the PROMIS-global health measure (4 items) [76] will also be assessed with the reference “TODAY.”

### Analytic Plans

We will first conduct extensive descriptive analyses of the retrospective and daily surveys over the 2-year period. Descriptive statistics, for example, means, SDs, and ranges for continuous measures, as well as frequencies and proportions for categorical variables, for the time-varying variables, will be calculated for each burst and for the 2-year period as a whole. Those of the baseline, person-level stable variables (eg, sex) will be calculated once. Preliminary bivariate analyses of the time-varying variables (eg, stress, health behaviors, and QoL) will be performed using within- and between-person correlations between them for each burst and for the 2-year period as a whole. Both correlations between an individual’s own variables (eg, individuals’ own stress and their health behaviors) and correlations between partners’ and survivors’ variables (eg, partners’ stress and survivors’ health behaviors) will be calculated.

For the aims of this study, we will design and fit mixed-effects multilevel models that adapt to the correlation structures of the underlying data process. For continuous (or near-continuous) outcomes (physical activity, eating behaviors, sleep, and QoL), linear mixed-effects models will be considered first, but depending on the distributional properties of the outcome (eg, skewed and censored), generalized linear mixed-effects models with proper distributions such as gamma or censored normal distribution and link functions such as log link will be used accordingly. For categorical outcomes (smoking and alcohol use), a logistic link function and binomial distribution will be used to build mixed-effects logistic regression models. All the models will be multilevel, incorporating burst-level trends, general day-to-day trends (centered), and deviations from the overall mean and day-to-day trends specific to burst (level 2) and to person (level 3) using random effects. Predictors will include survivors’ and partners’ day-to-day variables (person-burst-centered) to estimate within-person effects at level 1 and their burst-level variables (person-centered) to estimate within-person effects at level 2 (eg, the effect of day- or burst-level stress on health behaviors in Aim 1 and the effect of day- or burst-level health behaviors on QoL in Aim 2), their person-level (averaged) variables to estimate between-person effects at level 3, and a role variable to differentiate survivors’ and caregivers’ effects in each of those individuals’ own effects and partners’ effects using interaction terms. For the exploratory aims, 2-way interaction terms between the person-level variables (eg, person-level stress) and the day- or burst-level variables (eg, daily fluctuations of stress), as well as between-level stable demographic and clinical factors and the day- or bust-level variables, will be added to assess moderations for the individuals’ day- or burst-level effects. Three-way interaction terms with the role variable added will be used to differentially evaluate each of those 2-way interactions for survivors and partners.

Within this modeling framework, the components of the aims can be estimated and evaluated. For Aim 1, various components of stress will be related to concurrent health behaviors, both within and between individuals and across survivors and partners. The effect of interest is the association between stress components and individual health behaviors (at the burst level and the day level within bursts). Smoking and alcohol use will each be assessed as an indicator of behavior (with a logistic link) and a magnitude of behavior (excluding nonsmokers and nondrinkers, respectively). Linear relationships will be tested, and if model diagnostics indicate a nonlinear fit, a smooth nonlinear association will be assessed. A similar construction will be considered for Aim 2, relating components of stress to QoL and components of behavior to QoL at the burst level and the day level within bursts. Similarly, for the exploratory aim, the effects of interest will be interactions of day- or burst-level stress and aggregate-scale person-level stress or person-level stable factors. For each model, there is a possibility of nonignorable dropout or mortality. To address issues of missingness, we will assume that loss to follow-up is missing at random. Within this context and under a small number of assumptions, the missingness due to death or dropout becomes missing at random and ignorable given the context of the model. The mixed-effects structure, in particular, is robust to many patterns of random missingness. With respect to mortality, we will implement the principal stratification approach [77], wherein participants are weighted by their propensity to survive the study period.

### Sample Size and Power Calculation

Assuming a 20% rate of dropout, we expect to have at least 120 dyads complete the study. Most of our assessments will be measured by correlation. With 120 dyads, we expect to have at least 80% power to detect relationships with a partial *R*^2^ of at least 0.26, which is a medium-large effect. A similar construction shows that the 80% detectable level of the partial *F* is 0.06, which is a small-to-medium effect. The partial *F* statistic is a scaled version of the partial *F* statistic in a nested analysis of variance comparison; the scaling factor is the ratio of the numerator’s and the denominator’s degrees of freedom [78]. As the modeling structure and relative effect sizes (and confidence bounds) will be of primary interest, we will not incorporate a multiple testing structure until the correlation between test statistics can be carefully assessed (which allows for less conservative testing criteria). The R library *pwr* was used in the calculation of these effect sizes.

### Ethical Considerations

This study was approved by MD Anderson’s institutional review board (Protocol 2021-0165). Written informed consent will be obtained from all participants. Study participant research data, which will be obtained for purposes of statistical analysis and scientific reporting, will not include the participant’s contact or identifying information. Rather, individual participants and their research data will be identified by a unique study identification number. Participants will receive gift cards as compensation for their time and participation. Each participant will receive up to a US $35 gift card to complete a 7-day daily survey and wear an accelerometer (ie, US $5 gift card per day). Of the 7 days, participants must complete surveys and wear the accelerometer for at least 3 days to receive the compensation. Participants will also receive an additional US $15 gift card for completing both nutritional assessments and receive a US $40 gift card for each retrospective survey that they complete.

## Results

We are currently recruiting participants, having initiated recruitment in January 2022. Recruitment and data collection are expected to conclude by December 2024 and December 2026, respectively. We expect to submit the main study results for publication in 2027.

## Discussion

The aim of the proposed study is to investigate associations between stress, health behaviors, and QoL at both within- and between-person levels over the transition to survivorship in young adult survivors and their partners. To date, much is unknown regarding associations between stress, health behaviors, and QoL among young adult survivors. In particular, to the best of our knowledge, there is currently no existing study that investigates these associations among both young adult survivors and their partners. This will also be the first study that determines the interdependence of health behaviors and QoL between young adult survivors and partners, exploring how their stress levels may either facilitate or hinder their own and each other’s health behaviors and QoL over the transition to survivorship.

This study has a few limitations. First, recruiting survivor-partner dyads, particularly survivors within a restricted age range (25-39 years old), can be a challenge. We will thoroughly review the recruitment progress, study refusal reasons, and work on addressing enrollment barriers. Second, participants’ compliance may be a concern in this intensive longitudinal study. Over the course of the study, we will carefully monitor survey responses and consider reducing the number of retrospective or daily assessments if noncompliance is salient. Finally, this study addresses stress, health behaviors, and QoL of young adult survivors who have partners. Thus, the results of this study may not be generalized to all young adult survivors.

Despite these limitations, this study is novel and promises to generate new knowledge. Specifically, using a sophisticated research design—measurement burst—this study will allow assessment of both within- and between-person level changes in those variables and their relationships [56,57]. If the results of this study could reveal whether and on what time scales higher stress levels or certain types of stressors are associated with unhealthy behaviors and poor QoL, interventions such as just-in-time adaptive intervention [79] might be considered based on the findings. The focus on the transition to survivorship is another strength. By following up with the survivors over the course of 2 years after the completion of their cancer treatment, we can identify the time points when stress, health behaviors, and QoL begin to change (increase or decrease) during the transition. This information will help us target the right time to intervene for this understudied survivor group. Furthermore, the proposed study seeks to shift current individual-focused research in young adult survivors to relationship-focused exploration as it focuses on both survivors and their partners. Thus, the results of this study may guide the creation of a developmentally appropriate couple-based psychosocial or behavioral intervention that improves both young adult survivors’ and their partners’ QoL.

## Abbreviations

LAYA-SRQL: Late Adolescence and Young Adulthood Survivorship-Related Quality of Life
PROMIS: Patient-Reported Outcomes Measurement Information System
QoL: quality of life
REDCap: Research Electronic Data Capture

## Data Availability

The data sets generated during this study are not publicly available because they include sensitive information but are available from the corresponding author on reasonable request.
